# Supplementary material for: Ketamine for critically ill patients with severe acute brain injury: Protocol for a systematic review with meta-analysis and Trial Sequential Analysis of randomised clinical trials
Source: PLoS One. 2021 Nov 15;16(11):e0259899. doi: 10.1371/journal.pone.0259899 (PMC8592463; doi:10.1371/journal.pone.0259899)
Supplement: S1 File — (DOC) [file pone.0259899.s002.doc]

**Supplemental material 1 – Search strategies**

**The Cochrane Central Register of Controlled Trials (CENTRAL; 2020, Issue 10) in the Cochrane Library**

#1 MeSH descriptor: [Ketamine] explode all trees

#2 (ketamin* or esketamin* or arketamin* or ketalar or calypsol or ketajet or ketanest or ketaset or tekam or vetalar or soon-soon or narkamon)

#3 #1 or #2

#4 MeSH descriptor: [Brain Injuries] explode all trees

#5 MeSH descriptor: [Critical Illness] explode all trees

#6 MeSH descriptor: [Critical Care] explode all trees

#7 MeSH descriptor: [Subarachnoid Hemorrhage] explode all trees

#8 MeSH descriptor: [Brain Ischemia] explode all trees

#9 MeSH descriptor: [Intracranial Aneurysm] explode all trees

#10 MeSH descriptor: [Hypoxia, Brain] explode all trees

#11 MeSH descriptor: [Cerebrovascular Disorders] explode all trees

#12 (brain near injur* or (cerebrovascular and (disease* or disorder*)) or ((subarachnoid or intracranial or intracerebral) and h?emorrhage) or ischemi* or ((critical or intensive) and (illness or care)))

#13 #4 or #5 or #6 or #7 or #8 or #9 or #10 or #11 or #12

#14 #3 and #13

**MEDLINE Ovid (1946 to )**

1. exp Ketamine/

2. (ketamin* or esketamin* or arketamin* or ketalar or calypsol or ketajet or ketanest or ketaset or tekam or vetalar or soon-soon or narkamon).mp. [mp=title, abstract, original title, name of substance word, subject heading word, floating sub-heading word, keyword heading word, organism supplementary concept word, protocol supplementary concept word, rare disease supplementary concept word, unique identifier, synonyms]

3. 1 or 2

4. exp Brain Injuries/

5. exp Critical Illness/

6. exp Critical Care/

7. exp Subarachnoid Hemorrhage/

8. exp Brain Ischemia/

9. exp Intracranial Aneurysm/

10. exp Hypoxia, Brain/

11. exp Cerebrovascular Disorders/

12. (brain adj injur* or (cerebrovascular and (disease* or disorder*)) or ((subarachnoid or intracranial or intracerebral) and h?emorrhage) or ischemi* or ((critical or intensive) and (illness or care))).mp. [mp=title, abstract, original title, name of substance word, subject heading word, floating sub-heading word, keyword heading word, organism supplementary concept word, protocol supplementary concept word, rare disease supplementary concept word, unique identifier, synonyms]

13. 4 or 5 or 6 or 7 or 8 or 9 or 10 or 11 or 12

14. 3 and 13

15. (randomized controlled trial or controlled clinical trial).pt. or clinical trials as topic.sh. or trial.ti.

16. (random* or blind* or placebo* or meta-analys*).mp. [mp=title, abstract, original title, name of substance word, subject heading word, floating sub-heading word, keyword heading word, organism supplementary concept word, protocol supplementary concept word, rare disease supplementary concept word, unique identifier, synonyms]

17. 14 and (15 or 16)

**Embase Ovid (1974 to )**

1. exp ketamine/

2. (ketamin* or esketamin* or arketamin* or ketalar or calypsol or ketajet or ketanest or ketaset or tekam or vetalar or soon-soon or narkamon).mp. [mp=title, abstract, heading word, drug trade name, original title, device manufacturer, drug manufacturer, device trade name, keyword, floating subheading word, candidate term word]

3. 1 or 2

4. exp brain injury/

5. exp critical illness/

6. exp intensive care/

7. exp subarachnoid hemorrhage/

8. exp brain ischemia/

9. exp brain hemorrhage/

10. exp intracranial aneurysm/

11. exp brain hypoxia/

12. exp cerebrovascular disease/

13. (brain adj injur* or (cerebrovascular and (disease* or disorder*)) or ((subarachnoid or intracranial or intracerebral) and h?emorrhage) or ischemi* or ((critical or intensive) and (illness or care))).mp. [mp=title, abstract, heading word, drug trade name, original title, device manufacturer, drug manufacturer, device trade name, keyword, floating subheading word, candidate term word]

14. 4 or 5 or 6 or 7 or 8 or 9 or 10 or 11 or 12 or 13

15. 3 and 14

16. Randomized controlled trial/ or Controlled clinical study/ or trial.ti.

17. (random* or blind* or placebo* or meta-analys*).mp. [mp=title, abstract, heading word, drug trade name, original title, device manufacturer, drug manufacturer, device trade name, keyword, floating subheading word, candidate term word]

18. 15 and (16 or 17)

**LILACS (Bireme) (1982 to )**

(ketamin$ or esketamin$ or arketamin$ or ketalar or calypsol or ketajet or ketanest or ketaset or tekam or vetalar or soon-soon or narkamon) [Words] and ((brain and injur$) or (cerebrovascular and (disease$ or disorder$)) or ((subarachnoid or intracranial or intracerebral) and h?emorrhage) or ischemi$ or ((critical or intensive) and (illness or care))) [Words]

**CINAHL (EBSCO*host*;)**

S16 S14 AND S15

S15 TX (random* or blind* or placebo* or meta-analys*)

S14 S3 AND S13

S13 S4 OR S5 OR S6 OR S7 OR S8 OR S9 OR S10 OR S11 OR S12

S12 TX (brain near injur* or (cerebrovascular and (disease* or disorder*)) or ((subarachnoid or intracranial or intracerebral) and h?emorrhage) or ischemi* or ((critical or intensive) and (illness or care)))

S11 MW Cerebrovascular Disorders

S10 MW Hypoxia, Brain

S9 MW Intracranial Aneurysm

S8 MW Brain Ischemia

S7 MW Subarachnoid Hemorrhage

S6 MW Critical Care

S5 MW Critical Illness

S4 MW Brain Injuries

S3 S1 OR S2

S2 TX (ketamin* or esketamin* or arketamin* or ketalar or calypsol or ketajet or ketanest or ketaset or tekam or vetalar or soon-soon or narkamon)

S1 MW Ketamine

**Science Citation Index Expanded (1900 to ) and** **Conference Proceedings Citation Index – Science (1990 to )**

#5 #4 AND #3

#4 TI=(random* or blind* or placebo* or meta-analys* or trial*) OR TS=(random* or blind* or placebo* or meta-analys*)

#3 #2 AND #1

#2 TS=(brain near injur* or (cerebrovascular and (disease* or disorder*)) or ((subarachnoid or intracranial or intracerebral) and h?emorrhage) or ischemi* or ((critical or intensive) and (illness or care)))

#1 TS=(ketamin* or esketamin* or arketamin* or ketalar or calypsol or ketajet or ketanest or ketaset or tekam or vetalar or soon-soon or narkamon)
